# Assessment of human resources capacity of pharmaceutical warehouses in Cameroon

**DOI:** 10.1186/2052-3211-7-S1-P9

**Published:** 2014-12-17

**Authors:** Samuel Ottu Ayuk, Ojong Ntane Agbor, Felix Tanyi

**Affiliations:** 1 South West Regional Fund for Health Promotion, Regional Pharmaceutical Supply Center, Yaoundé, Cameroon

## Background

Cameroon’s health system is based on a pyramidal model, with three levels: central, intermediate and peripheral. This report describes the key findings of the assessments of the human resources of the Central Medical Stores (CENAME) and the 10 regional pharmaceutical supply centres (CAPRs) operating the public supply chain for pharmaceuticals and health commodities in Cameroon. The central level consists of the MOH whose main role is to define national strategy and policy, as well as of national-level hospital. The intermediate level consists of 10 regional delegation of public health, which provides technical support, coordination, oversight and supervision of health districts. This level also includes regional hospitals. The main objective of this assessment was to develop an improvement plan aimed at strengthening the human resources capacity in the public pharmaceutical sector in Cameroon at national and regional level.

## Method

A tailor made tool was developed based on existing assessment tools, the World Health Organization good distribution practices for pharmaceutical products, and Cameroon’s Good distribution practices for health products, and adapted to the local context. Data collected during the field visits were analysed identifying the strengths, weaknesses and recommendations.

## Results

Most of the CAPRs have competent staff that are sufficient in number. Some CAPRs were overstaffed – mainly with regards to operational staff - relative to the current workload. It is not uncommon that a staff member occupies different positions at the same time. All CAPRs have at least one pharmacist and in most cases this is the manager. Three CAPRs have one or two additional pharmacists. CENAME had six pharmacists including the general manager. Organizational charts were found in nine of the CAPRs/CENAME, though in five CAPRs the versions available were outdated and very general. Seven CAPRs/CENAME provided their (warehouse) staff with special uniforms, though not all staff wear them. In at least five CAPRs/CENAME, some relevant staff were found not to have sufficient and/or workable knowledge of good storage and distribution practices. Many of the staff had basic background and knowledge required for their position. There is no training plan that takes into account the objectives of the CAPRs/CENAME.

## Discussion

Human resources plays a vital role in the supply chain management of medicines, thereby improving their capacities would result to improved quality of services.

## Lessons learned

The improvement plan is a first step towards improving human resources. Training is essential for an efficient workforce as currently adequately trained personnel are limited and are employed to work in areas where they are not competent. The unavailability of work based tools is also a challenge.

